# A new strategy for the fabrication of a flexible and highly sensitive capacitive pressure sensor

**DOI:** 10.1038/s41378-021-00327-1

**Published:** 2021-11-30

**Authors:** Ruzhan Qin, Mingjun Hu, Xin Li, Te Liang, Haoyi Tan, Jinzhang Liu, Guangcun Shan

**Affiliations:** 1 School of Instrumentation Science and Opto-electronics Engineering, Beihang University, Beijing, 100191 China; 2 School of Materials Science and Engineering, Beihang University, Beijing, 100191 China

**Keywords:** Sensors, Electrical and electronic engineering

## Abstract

The development of flexible capacitive pressure sensors has wide application prospects in the fields of electronic skin and intelligent wearable electronic devices, but it is still a great challenge to fabricate capacitive sensors with high sensitivity. Few reports have considered the use of interdigital electrode structures to improve the sensitivity of capacitive pressure sensors. In this work, a new strategy for the fabrication of a high-performance capacitive flexible pressure sensor based on MXene/polyvinylpyrrolidone (PVP) by an interdigital electrode is reported. By increasing the number of interdigital electrodes and selecting the appropriate dielectric layer, the sensitivity of the capacitive sensor can be improved. The capacitive sensor based on MXene/PVP here has a high sensitivity (~1.25 kPa^−1^), low detection limit (~0.6 Pa), wide sensing range (up to 294 kPa), fast response and recovery times (~30/15 ms) and mechanical stability of 10000 cycles. The presented sensor here can be used for various pressure detection applications, such as finger pressing, wrist pulse measuring, breathing, swallowing and speech recognition. This work provides a new method of using interdigital electrodes to fabricate a highly sensitive capacitive sensor with very promising application prospects in flexible sensors and wearable electronics.

## Introduction

Wearable devices have wide application prospects in human-computer interfaces, soft robots, health monitoring and electronic skin^1^. With the increasing demand for wearable devices, high-performance flexible pressure sensors have attracted wide attention. Pressure sensors can be divided into piezoresistive^2^, piezoelectric^3^, capacitive^4^ and triboelectric effect sensors^5,6^. Capacitive sensors have become the focus of current research due to their fast dynamic response, excellent temperature insensitivity and low power consumption^7^. Although pressure sensors have made great progress in the past few years, there are still many challenges regarding the fabrication of highly sensitive sensors. In particular, flexible capacitive pressure sensors generally have the characteristics of low sensitivity^8–17^. Most capacitive pressure sensors are fabricated as parallel plate capacitors, which are characterized by a dielectric layer sandwiched between two electrode plates^8,10,12,14–16,18–23^. However, a capacitive sensor with a parallel plate structure is difficult to miniaturize and achieve ultrathinness; additionally, the upper and lower electrode structures are not easy to design and integrate. Traditional parallel plate capacitive pressure sensors usually have the disadvantage of low sensitivity^8,10,12,14–16^. Therefore, a lot of efforts need to be made to find a flexible pressure sensor with a high sensitivity, fast response time and low detection limit to meet the needs of wearable applications.

To find a better performing capacitive pressure sensor, scientists have made great efforts. By increasing the dielectric constant or changing the geometric structure of the dielectric layer, such as fabricating a dielectric layer with a porous or foam-like dielectric layer and a microstructure dielectric layer, the elastic modulus can be reduced to improve the sensitivity of the sensor^10,14,15,17,20^. According to the formula:

1 $$C = \frac{{\varepsilon _0\varepsilon _rA}}{d}$$

*C* is the capacitance value, *ε*_*0*_ is the vacuum permittivity, *ε*_*r*_ is the relative permittivity, *A* is the facing area of the upper and lower electrodes and *d* is the distance between the electrodes^10^. The methods of causing capacitance change mainly include the change in the dielectric constant of a dielectric layer, the distance between two electrode layers and their facing area^13^, while the first two parameters can be adjusted by deformation of the dielectric layer with porous and foam-like structures. The porous and foam-like structures can add some air into the dielectric layer during deformation, which increases its dielectric constant and easily changes the distance between the two electrode layers. This is a common method to improve the sensitivity of a sensor^11,14,15,21,22^. Although the elastic modulus of a dielectric layer can be reduced by the use of a porous structure, it has a low capacitance value due to its low dielectric constant.

The other strategy to improve the sensitivity is to reduce the elastic modulus by introducing a microstructure into the dielectric or electrode layer instead of a conventional flat layer. The microstructure of the dielectric layer or electrode layer includes a rough interface, microcolumn array, circular convex array and micropyramid^8–10,13,16,20,23–25^. The microstructure of the dielectric layer or electrode layer also includes an electrode array, lotus leaf and rose petal microstructure and rough paper^25–27^. Unfortunately, the microstructure usually requires complex and time-consuming manufacturing processes such as photolithography or chemical etching, which is unsuitable for large-scale production and application.

Increasing the dielectric constant is an ideal way to achieve a high initial capacitance and large capacitance variations. The commonly used dielectric layers are PDMS, Ecoflex and PET films^8,10,12–16,24,28^. Generally, composite materials with high dielectric constants can be used instead of materials with low dielectric constants. However, increasing the dielectric constant of a dielectric layer may increase its thickness, which is not conducive to its miniaturization and integration in a sensor. In addition, the increase in the dielectric constant is also limited. Because the current method has some defects, we need a better method to achieve a capacitive pressure sensor with high performance.

To date, increasing the number of interdigital electrodes combined with soft dielectric layers has not been reported to improve the sensitivity of capacitive sensors. The two-dimensional nanomaterial MXene is undoubtedly an ideal sensor material because of its abundant active catalytic sites (OH, F, O), large specific surface area, good dispersion and high ion intercalation^29–31^. Compared with the characteristics of other nanomaterials, these characteristics of MXene result in effective ionic constraints on the material surface. Thus, MXene is introduced into the dielectric layer to produce an ion limiting effect. Due to this effect, positive and negative ion pairs are connected to the functional groups of MXene sheets embedded in PVP. The use of MXene is conducive to the dynamic constraint of ions under external stimulation and affects the capacitance characteristics of highly sensitive pressure sensors over a wide linear range. In this work, we demonstrate a high-performance flexible capacitive sensor using interdigital electrodes combined with an MXene/PVP dielectric layer. The experimental results show that increasing the number of interdigital electrodes has a good effect on improving the sensitivity. The capacitive sensor based on MXene/PVP shows short response and relaxation times (30/15 ms), a high pressure sensitivity (1.25 kPa^–1^), good repeated loading stability (10000 cycles) and a low detection limit (0.6 Pa). As a proof of concept, the MXene/PVP-based sensor can be used to detect pulse, voice, breathing and swallowing signals, and a 3 × 3 sensor array is used to map the pressure distribution of different objects. This work provides a new idea and strategy for the preparation of highly sensitive capacitive flexible sensors.

## Results and discussions

### Fabrication of the MXene/PVP-based capacitive pressure sensors

**Fig. 1:**
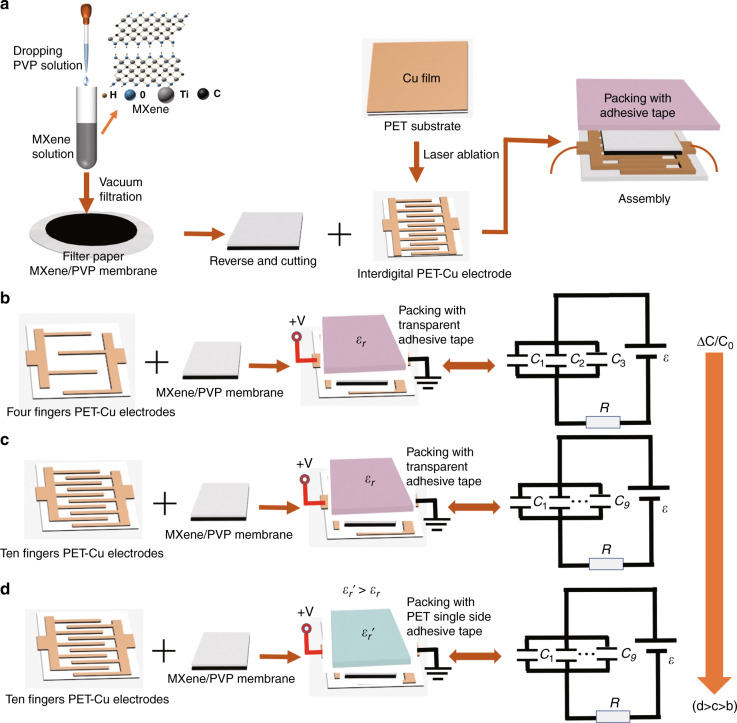
Schematic illustration of the fabrication process and sensing mechanism of the MXene/PVP-based flexible pressure sensors. **a** Schematic illustration of the fabrication process of the MXene/PVP-based capacitive pressure sensor. **b**–**d** Sensing mechanism and equivalent circuit of the MXene/PVP-based capacitive pressure sensor

Figure 1a shows the preparation process of the flexible capacitive pressure sensor based on MXene/PVP and interdigital electrodes. Traditional capacitive sensors consist of two electrode layers separated by insulating materials. The structure of the traditional capacitive sensor is different from ours, with the top layer being PET single-sided adhesive tape as the packaging layer, the middle layer being an MXene/PVP filter paper membrane as the sensitive layer and the bottom layer being a copper interdigital electrode on a flat polyethyleneterephthalate (PET) substrate as the output signal layer. The PET-Cu interdigital electrode layer was prepared by the previously reported laser ablation process^32^. Figure S1 (Supplementary Information) shows a photograph of the PET-Cu interdigital electrode prepared by the laser ablation strategy. The size of the PET-Cu interdigital electrode is designed to be 1.5 cm × 1 cm with 12 fingers. Interdigital electrodes with 12 fingers are used to prepare sensors assembled with different dielectric layers for the performance test. Additionally, a sensor array is prepared by using interdigital electrodes with 12 fingers. The depth and width of the fingers are 5 mm and 300 μm, respectively. Figure S2a–f (Supplementary Information) shows photographs of the PET-Cu interdigital electrodes with different numbers of interdigital fingers and the optical microscope images of the PET-Cu interdigital electrodes with 4, 6, 10, 14 and 44 fingers, respectively. Figure S3 (Supplementary Information) shows the front, back and cross-section of the prepared sensor (44 fingers). The cross-sectional view of the sensor is compared with the paper thickness, and the thickness of the sensor is ~400 µm. The preparation method of MXene can be seen in the Experimental section. A small amount of PVP is added to the prepared MXene aqueous solution to adjust the resistivity of the MXene. Then, the mixture of MXene and PVP is filtered in a vacuum to form a film. The MXene/PVP membrane is attached to filter paper as a dielectric layer. Then, a flexible capacitive sensor is fabricated by simple assembly, as shown in Fig. 1a.

### Sensing mechanism of the MXene/PVP-based capacitive pressure sensors

A capacitive sensor is a device that converts a pressure change into a capacitance change; in other words, the change in pressure is observed by outputting a capacitance signal. According to Formula (1), the capacitance can be changed by changing the *ε*_*r*_ of the dielectric layer, area *A* of the two plates and distance d between the two plates. The sensor is prepared by selecting the appropriate dielectric layer, and the change in capacitance is changed by the deformation of the dielectric layer. As shown in Fig. 1b, we selected four of PET-Cu electrode fingers as the signal acquisition layer, and then used an MXene/PVP filter paper layer as the dielectric layer and a PET single-sided adhesive tape layer (thickness of ~390 µm) as the sealing layer to prepare a capacitive pressure sensor. The prepared sensor has a certain initial capacitance value (Table 1). When the sensor is under a certain pressure, the dielectric layer of the sensor will be deformed by the force, which will lead to a change in the dielectric constant of the dielectric layer and the facing area between the two electrodes. Due to the gap between the MXene/PVP layer and PET-Cu electrode layer, the MXene/PVP layer only partially contacts the PET-Cu electrode layer. With increasing pressure, the gap between layers decreases, and thereby the MXene/PVP layer comes into close contact with the PET-Cu electrode layer. Moreover, because of the concave and convex surface of the filter paper, the MXene/PVP membrane layer is concave and convex, which also increases the gap between the MXene/PVP layer and PET-Cu electrode layer. Figure S2b–f shows that there are grooves between the fingers of the interdigital electrode. When the sensor is under pressure, the dielectric layer will fill the grooves between the fingers of the interdigital electrode due to deformation, which will increase the capacitance change of the sensor, as shown in Fig. S4a, b (Supplementary Information). When the sensor has no loading pressure, the gap between the two Cu electrode pairs is air. In contrast, when the sensor is under a loading pressure, part of the MXene/PVP dielectric layer fills the gap between the two electrode pairs. The greater the loading pressure is, the more the dielectric layer fills the gaps. This results in an increase in the dielectric constant between the two electrodes, which leads to an obvious change in capacitance. In addition, an interdigital electrode with multiple fingers is equivalent to a parallel configuration of multiple sensors, which will greatly increase the capacitance variation of the sensor. As shown in Fig. 1b, the sensor composed of 4 interdigital PET-Cu electrode fingers, an MXene/PVP filter paper membrane and a transparent adhesive tape layer (thickness of ~50 µm) is equivalent to a sensor composed of 3 capacitors *C*_1_, *C*_2_ and *C*_3_ in parallel. The circuit diagram of the sensor is shown in the equivalent circuit on the right side of Fig. 1b, in which the copper electrode resistance *R* is considered in the circuit. There is a certain edge effect capacitance in the overall sensor capacitance, but the edge effect capacitance is small and can be ignored.

**Table 1:** Capacitance changes of MXene/PVP-based sensors with different numbers of interdigital electrode fingers and an MXene/PVP filter paper membrane with ~50 kΩ/sq resistivity

Number of fingers	Capacitance variation range
4	1–30 pF
6	3–100 pF
10	3–130 pF
14	4–300 pF
44	6–750 pF

To improve the capacitance variation, we used an interdigital electrode with 10 fingers instead of an interdigital electrode with 4 fingers, an MXene/PVP filter paper membrane with the same size and thickness as the dielectric layer, and packaged the setup with transparent adhesive tape. Notably, the initial capacitance changes little, but under a certain pressure, the capacitance changes more obviously, indicating that an increase in the number of fingers significantly improves the capacitance change of the sensor. As shown in Fig. 1c, the sensor composed of 10 interdigital PET-Cu electrode fingers, an MXene/PVP filter paper membrane and a transparent adhesive tape layer is equivalent to a sensor composed of 9 capacitors *C*_1_, *C*_2_, … and *C*_9_ in parallel. On the basis of the above sensor, we replaced the transparent adhesive tape layer (thickness of ~50 µm) with a PET single-sided adhesive tape layer (a low modulus, thickness of ~390 µm) because it has a higher dielectric constant. The capacitance changes more obviously under a certain pressure because the packaging material with a lower compression modulus deforms more under a given applied pressure. This shows that choosing an appropriate packaging layer can also greatly improve the change in capacitance, as shown in Fig. 1d. The more deformation the dielectric material experiences under a given pressure, the greater the change in its capacitance; thus, its sensitivity increases. From Fig. 1b–d, it is found that the change in ΔC/C_0_ increases from top to bottom (d > c > b).

It can be seen from the above that selecting the MXene/PVP filter paper as the dielectric layer or increasing the number of fingers of the interdigital electrode can increase the change in capacitance under a certain pressure. It can also increase the change in capacitance by selecting an appropriate dielectric layer (*ε*_*r*_*’ >ε*_*r*_) for upper surface packaging, which increases the dielectric constant of the whole dielectric layer (MXene/PVP filter paper membrane + PET single-sided adhesive tape layer). Unless otherwise stated, the sensors prepared later are encapsulated with PET single-sided adhesive tape.

### Structure and characterization of the MXene/PVP-based capacitive pressure sensors

**Fig. 2:**
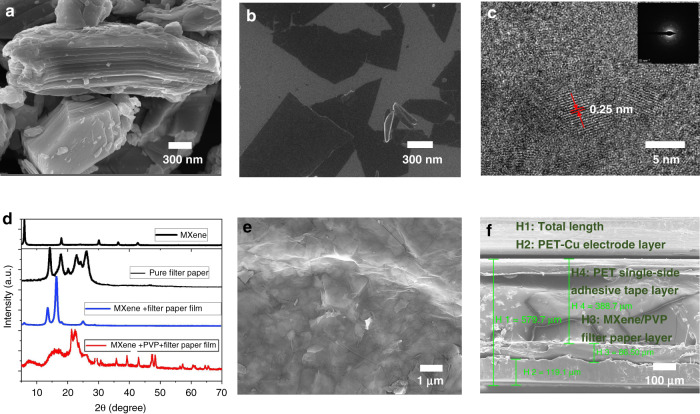
Characterization of the MXene and MXene/PVP-based capacitive pressure sensors. **a** SEM image of MAX (Ti_3_AlC_2_) phase. **b** SEM image of MXene (Ti_3_C_2_T_x_). **c** High-resolution TEM image of MXene and its corresponding diffraction pattern (in the inset). **d** X-ray diffraction patterns of pure filter paper, MXene, MXene filter paper film and MXene/PVP filter paper film. **e** SEM image of the MXene/PVP filter paper surface. **f** SEM image of the cross-sectional structure of the MXene/PVP-based capacitive pressure sensor

The selection of MXene with various layer spacings as the dielectric layer material of a capacitive sensor is mainly based on the following considerations. First, MXene is hydrophilic and easy to disperse in water. Polymers can be added to adjust the resistivity of MXene. Second, there are abundant functional groups (-O, -F, -OH) on the surface of MXene, and the surface is negatively charged, which has a certain regulating effect on the charge distribution of the dielectric layer in the capacitive sensor. Ti_3_C_2_T_x_ MXene is obtained by selectively etching element “A” from the MAX phase of Ti_3_AlC_2_ (Fig. 2a, b). The planar high-resolution TEM image of MXene and its corresponding diffraction pattern (in the inset) show that the interlayer distance of MXene is approximately 0.25 nm (Fig. 2c). Figure 2d shows XRD images of the fresh MXene, pure filter paper, Mxene filter paper film and Mxene/PVP filter paper film. Figure 2d shows that most of the Ti_3_AlC_2_ is successfully transformed into Ti_3_C_2_T_x_. The SEM image of the MXene/PVP membrane surface is shown in Fig. 2e, and some MXene sheet structures can be seen on the membrane surface. The surface of the MXene/PVP filter paper membrane is uneven, as shown in Fig. 2e, because the surface of the filter paper is uneven and porous, as shown in Fig. S5 (Supplementary Information). The surface of the filter paper has a rough interface with microscale characteristics (Fig. S5), so that the MXene/PVP filter membrane attached to the surface of the filter paper also has a rough surface. The air gap at the interface is used to improve the compressibility and capacitive sensitivity. The cross-section of the sensor is shown in Fig. 2f, indicating that the thickness of the sensor device is 579.7 µm. The thicknesses of the PET-Cu layer, MXene/PVP filter paper membrane layer and PET single-sided adhesive tape of the sensor are 119.1 µm, 86.5 µm and 388.7 µm, respectively. The thickness of the MXene/PVP filter film layer is ~4 µm, as shown in Fig. S6 (Supplementary Information). The surface element mapping and energy spectrum of the MXene/PVP filter film are shown in Fig. S7 (Supplementary Information). Due to the porous and uneven surface structure of the filter paper, the contact area between the MXene/PVP filter paper membrane and PET-Cu interdigital electrode increases slowly under force, resulting in an obvious change in sensor capacitance.

### Performance of the MXene/PVP-based capacitive pressure sensors

To verify the feasibility of realizing high-performance capacitive sensors by increasing the number of interdigital electrodes, we compare and discuss the performance of sensors prepared with different numbers of interdigital electrodes, and the effectiveness of this method for improving the change in capacitance change is analyzed. The sensitivity and sensing range of the sensor are the two most important performance indicators of a sensor. The improvement in sensitivity is mainly realized by increasing the change in the capacitance within a certain pressure range. Thus, we tested various design ideas. We used an interdigital PET-Cu electrode (12 fingers) and PET single-sided adhesive tape to fabricate a capacitive sensor without a MXene/PVP filter paper membrane. Then, we used a pressure testing device and source meter to test the performance of the sensor in the pressure range of 130.66 Pa–294 kPa, as shown in Fig. S8 (Supplementary Information). The output capacitance of the capacitive sensor made of only the interdigital electrode and PET single-sided adhesive tape does not change regularly in this pressure range, as shown in Fig. S9 (Supplementary Information). Then, we used an interdigital PET-Cu electrode (12 fingers), pure filter paper and PET single-sided adhesive tape to prepare capacitive sensors without an MXene/PVP membrane. Again, the capacitance changes irregularly in the pressure range of 130.66 Pa–294 kPa, as shown in Fig. S10 (Supplementary Information). It can be concluded that as long as there is no MXene/PVP filter paper membrane layer as a dielectric layer. The capacitance change of the sensor is irregular in the pressure range of 130.66 Pa–294 kPa. Fig. S11 (Supplementary Information) shows that the capacitance change of a sensor encapsulated with 3 M double-sided tape (without an MXene/PVP filter paper membrane) is irregular in a pressure range of 130.66 Pa–294 kPa. Fig. S12 (Supplementary Information) shows that the capacitance change of a sensor assembled with an MXene/polyvinyl alcohol (PVA) filter paper membrane (~50 kΩ/sq) and encapsulated with PET single-sided adhesive tape is regular in the pressure range of 130.66 Pa–294 kPa. The capacitance of the sensor varies from 5 pF to 110 pF. Figure S13 (Supplementary Information) shows that the capacitance change of a sensor assembled with an MXene/PVP filter paper membrane (~50 kΩ/sq) and without a PET single-sided adhesive tape layer (PET-Cu electrode with 12 fingers) is regular in the pressure range of 130.66 Pa–294 kPa. The capacitance of the sensor varies from 5 pF to 85 pF. However, the capacitance change is not obvious at low pressure. The performance of sensors with the PET single-sided adhesive tape is better than those without it, which can be confirmed by later experiments.

For the convenience of comparison and understanding, the performance test and analysis of the following sensors are based on 16 sampling points between 130.66 Pa and 294 kPa: 130.66 Pa, 326.66 Pa, 653.33 Pa, 1.31 kPa, 3.27 kPa, 6.53 kPa, 13.07 kPa, 32.67 kPa, 65.33 kPa, 98 kPa, 130.67 kPa, 163.33 kPa, 196 kPa, 228.67 kPa, 261.33 kPa and 294 kPa. Additional sampling points are selected for testing, and there are obvious regular changes at each sampling point, which shows that the sensor has good resolution. Each sampling point is tested for two consecutive cycles. We used an interdigital PET-Cu electrode with 12 fingers, a pure MXene filter paper membrane as a dielectric layer and PET single-sided adhesive tape to prepare a capacitive sensor. The conductivity of the MXene filter membrane is excellent, and the capacitive sensor assembled with the interdigital electrode fails due to a short circuit. To use MXene as a dielectric layer, we added an appropriate amount of PVP polymer (see the Experiment section) into the MXene aqueous solution to adjust the resistivity of MXene. We used an interdigital PET-Cu electrode with 12 fingers, an MXene/PVP filter paper membrane (~1.5 kΩ/sq) and PET single-sided adhesive tape to prepare capacitive sensors and determined that the capacitance changed irregularly in a certain pressure range of 130.66 Pa–294 kPa, as shown in Fig. S14 (Supplementary Information). An MXene/PVP filter paper membrane with a resistivity of ~30 kΩ/sq was then used to replace the ~1.5 kΩ/sq MXene/PVP filter paper membrane. We find that the capacitance of the sensor changes regularly in a certain pressure range of 130.66 Pa–294 kPa, as shown in Fig. S15 (Supplementary Information). However, the variation in capacitance in the low-pressure region is not obvious. At this point, the capacitance of the sensor changes from ~6 pF to ~120 pF in the pressure range of 130.66 Pa–294 kPa. An MXene/PVP filter paper membrane with a resistivity of ~50 kΩ/sq was used to replace the ~30 kΩ/sq MXene/PVP filter paper membrane. We find that the capacitance of the sensor changes more obviously in a certain pressure range of 130.66 Pa–294 kPa, as shown in Fig. S16 (Supplementary Information). At this point, the capacitance of the sensor changes from ~3 pF to ~205 pF in the pressure range of 130.66 Pa–294 kPa. Next, an MXene/PVP filter paper membrane with resistivity of ~3.9 MΩ/sq was used to replace the ~50 kΩ/sq MXene/PVP filter paper membrane. We find that the capacitance variation of the sensor is not as obvious in the pressure range of 130.66 Pa–294 kPa. The change in capacitance of the sensor is also smaller (~2 pF to ~25 pF) in the pressure range of 130.66 Pa–294 kPa, as shown in Fig. S17 (Supplementary Information). From the above analysis, it can be seen that adding an appropriate amount of PVP to the MXene material increases the resistivity of the mixed film, resulting in obvious changes in the capacitance of the sensor. However, excess PVP will reduce the capacitance change of the sensor, because excess PVP will make it difficult to separate the positive and negative ions on the MXene sheets. Furthermore, it is found that an appropriate dielectric layer can be obtained by combining an appropriate amount of PVP to MXene, which is an important factor to obtain an obvious change in sensor capacitance. The number of interdigital electrodes used in the preparation of the above sensors was 12 pairs. In the next section, we further study the performance of sensors assembled with different numbers of interdigital fingers.

We used an MXene/PVP filter paper membrane with a resistivity of ~50 kΩ/sq to prepare capacitive sensors with various numbers of PET-Cu interdigital electrodes l fingers. Figure S18 (Supplementary Information) shows the capacitance curves of the capacitive sensors with an MXene/PVP filter paper membrane of ~50 kΩ/sq, PET single-sided adhesive tape and various numbers of interdigital electrode fingers in the pressure range of 130.66 Pa–294 kPa. Figure S18a–o correspond to the interdigital electrodes with 4, 6, 10, 14 and 44 interdigital fingers, respectively. As shown in Fig. S18, the capacitance changes of the sensors with different numbers of interdigital fingers are different. The more fingers there are, the greater the capacitance changes, as shown in Table 1.

**Fig. 3:**
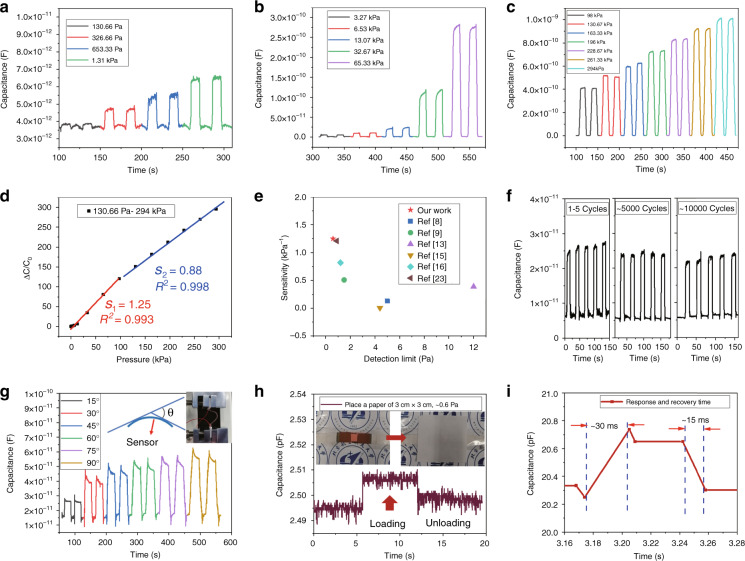
Performance of the capacitive pressure sensors based on MXene/PVP. **a**–**d** *C*−*T* curves at different pressures and their corresponding sensitivities. **e** Comparison between our capacitive pressure sensor and previously reported MXene-based and other material capacitive pressure sensors with sensitivities and detection limits. **f** Stability tests of more than 10,000 cycles of loading and unloading 6.53 kPa. **g** *C*−*T* curves of the capacitive pressure sensor based on MXene/PVP under different bending angles. **h** Low-pressure detection of the MXene/PVP sensor by placing a 5 cm × 5 cm sheet of paper (0.1607 g) shows that the limit of detection is 0.6 Pa. **i** Response and recovery times of the sensor based on MXene/PVP

From the above data analysis in Table 1, it can be seen that increasing the number of interdigital electrode fingers can increase the change in capacitance. However, there is also a problem; that is, when a large number of PET-Cu interdigital electrodes of the same size are selected, the gap between two interdigital pairs is very small, resulting in a small capacitance value between the two electrode pairs; this results in little change in the capacitance of the sensor under the same pressure. Therefore, choosing the appropriate number of interdigital electrodes is another important factor to obtain a large capacitance change. The sensing performance of the pressure sensor based on the MXene/PVP filter paper membrane can be improved by changing the geometry and dielectric constant. To improve the sensitivity of the capacitive sensor, an effective method is to reduce the initial capacitance of the sensor so that the sensitivity will be higher. When the transparent adhesive tape (*ε*_*r*_) is replaced by PET single-sided adhesive tape (*ε*_*r*_*’*) with a small elastic modulus, the dielectric constant of the capacitive sensor clearly changes, and the capacitance changes more obviously. This benefits from the coverage of PET single-sided adhesive tape with viscous paste, which increases the deformation of the dielectric layer. Fig. S19 (Supplementary Information) shows the SEM image and the locally enlarged images of the surface structure of PET single-sided adhesive tape with a small elastic modulus. A higher sensitivity is obtained by optimizing the number of interdigital electrode fingers (36 fingers), selecting PET single-sided adhesive tape with a better dielectric constant and reducing the contact area of MXene/PVP. As shown in Fig. 3a-c, the capacitance increases slowly and regularly with increasing loading pressure, which shows that the sensor has good resolution. The initial capacitance of the optimized sensor is ~3 pF, and the maximum capacitance is ~1000 pF at 294 kPa.

The sensitivity of the capacitive sensor is defined as:

2 $$S = \frac{{(C - C_0)/C_0}}{{\Delta P}} = \frac{{\Delta C/C_0}}{{\Delta P}}$$

where *C* is the instantaneous capacitance value of the sensor when there is a load, *C*_0_ is the initial capacitance value without a load, and Δ*P* is the change in applied pressure. If the initial loading pressure is 0, then Δ*P* = *P* (*P* is the applied pressure). The sensitivity of the capacitive sensor is 1.25 kPa^−1^ in the pressure range of 130.67 Pa-98 kPa and 0.88 kPa^−1^ in the pressure range of 98 kPa-294 kPa, as shown in Fig. 3d. The sensitivity can reach as high as 1.25, which is significantly higher than those of MXene-based or other material capacitive pressure sensors reported in the last three years^8,9,13,15,16,23^ (Fig. 3e). Table S1 (Supplementary Information) summarizes and compares the sensing properties of capacitive pressure sensors based on MXene or other materials that have been reported in the last few years^8,9,11,13,15–17,23,33^. Figure 3f shows the cycling stability of the MXene/PVP-based capacitive pressure sensors. After 10000 compression loading/unloading tests of 6.53 kPa, the capacitance output of the sensor decreases due to internal fatigue caused by long-term pressure loading; however, this is still representative of good stability. To show the flexibility of our sensor, we performed a bend test. The sensor can output a regular capacitance variation curve at different bending angles, which shows that the sensor has a certain flexibility, as shown in Fig. 3g. Figure 3h shows that the output of the capacitance value changes after loading and unloading 5 cm × 5 cm paper (0.1607 g), indicating that the limit of detection of the sensor is ~0.6 Pa (Movie S1, Supplementary Information). Figure 3i shows that the response time and recovery time of our sensor are 30 ms and 15 ms, respectively. This is much faster than the response times of many sensors^8,9,11,13,15–17,23^. Figure S20 (Supplementary Information) shows the frequency response curve of the sensor. Notably, the capacitance output performance of the sensor is good at different frequencies.

### Applications of the MXene/PVP-based capacitive pressure sensor

**Fig. 4:**
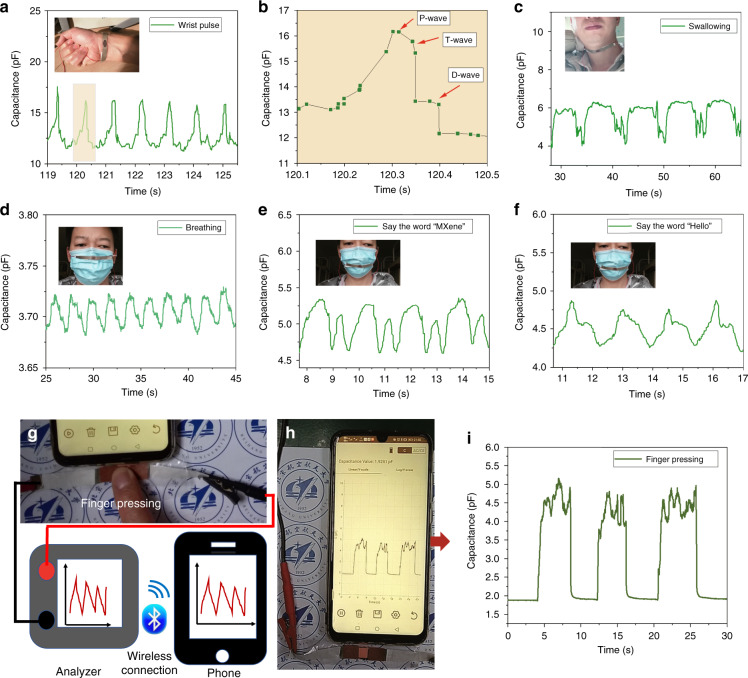
Application test of the capacitive pressure sensors based on MXene/PVP. **a** The sensor is attached to the wrist to sense the arterial pulse. **b** Magnified view of the wrist pulse waveform showing characteristic peaks (P-wave, T-wave, and D-wave). **c** The sensor is attached to the throat to sense the swallowing action of the human body. **d** The sensor is attached to the nose of the mask to sense the breathing of the human body. **e**, **f** The sensor is attached to the mouth of the mask to test speech recognition. **g** The real response of finger pressing is displayed on the mobile phone through wireless transmission. **h**, **i** *C*-*T* curve of the finger pressing sensor displayed on the mobile phone

From the above analysis, we can see that our capacitive sensors have good performance and wide application prospects in regard to wearable electronics and other fields. To demonstrate the practicability of our proposed sensor in the field of human health monitoring, we attached the sensor to a wrist for pulse signal acquisition. The regular pulse signal can be seen in Fig. 4a. The magnified view of the wrist pulse waveform in Fig. 4b shows the characteristic peaks of the P-wave, T-wave and D-wave. We attached the sensor to the throat for a swallowing test. As shown in Fig. 4c, the sensor can detect a clear swallowing signal. At present, COVID-19 is prevalent worldwide. Wearing masks can prevent the spread of the virus. Therefore, we attached sensors to masks for a breathing test, and the sensor can clearly distinguish the changes in capacitance signals from normal breathing (Fig. 4d). This shows that our sensor can be applied to monitoring the breathing of patients. The sensor can also be attached to the front of the mouth of the mask for voice detection. Figure 4e, f shows the change in capacitance when we say “MXene” and “hello”, indicating that our sensor can be used in the field of speech recognition. Figure 4g–i shows the real-time wireless sensing performance of the pressure sensor based on MXene/PVP during finger pressing. The finger pressing response of the capacitive sensor presented here can also be observed in Movie S2 (Supplementary Information). Especially in recent years, the rapid development of nanogenerators has made wearable self-powered health monitoring devices with wireless transmission convenient^5,34–36^. The integration of nanogenerators and capacitive sensors will be receiving more attention and application prospects^35^.

**Fig. 5:**
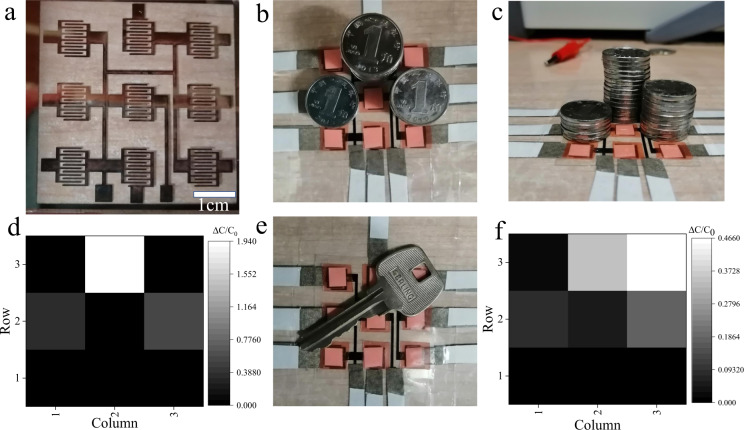
Application test of the capacitive sensor array based on MXene/PVP. **a** Interdigital electrode and circuit of the 3 × 3 sensor array. **b**, **c** The coins with a value of 0.1 RMB and quantities of 5, 10 and 15 are stacked in different positions of the 3 × 3 sensor array. **d** Distribution of the position and pressure of the sensor array corresponding to (**b**) and (**c**). **e** The response is achieved by placing a key on the sensor array. **f** The distribution of the position and pressure of the corresponding (**e**).

Capacitive sensors are widely used in electronic skin and human-computer interactions. We have prepared 3 × 3 sensor arrays to achieve spatial information acquisition and pressure distribution mapping, as shown in Fig. 5. The PET-Cu sensor array was fabricated by laser ablation^29^. MXene/PVP filter paper membranes were used to cover each sensor electrode unit, and PET single-sided adhesive tape was used to package each sensor unit. Each component of the sensor array was an independent sensor unit. Coins with a value of 0.1 RMB and quantities of 5, 10 and 15 were stacked in different positions on the array, as shown in Fig. 5b, c. The pressure of each position was measured, and the distribution of the positions and intensities of the test pressures are shown in Fig. 5d. In addition, we also put a key on the sensor array, as shown in Fig. 5e. The capacitance of each unit of the sensor array was tested, and the distribution of the pressure positions and intensity of the test pressure are shown in Fig. 5f. From the pressure mapping in Fig. 5d-f, it can be seen that the portable flexible capacitive sensor based on MXene/PVP is suitable for use in wearable devices and electronic skin.

## Materials and methods

### Materials

MAX phase (Ti_3_AlC_2_) powder (200 mesh) and LiF (98%) were purchased from Beijing Wisdom Company and Aladdin Reagent Company of China, respectively. Hydrochloric acid (36–38%) was purchased from Beijing Chemical Works of China. PVP was purchased from Shanghai Aladdin Industrial Corporation of China. A copper-clad PET film (200 nm-thick copper coating layer and 100 µm-thick PET substrate) was purchased from a commercial company (Teonex Q65FA, Graphene Testing and Sales of Platform Co., Ltd., China). Deionized water (DI, 18 MΩ) was used to prepare all solutions.

### Preparation of the MXene and MXene/PVP filter paper membranes

LiF (1 g) was slowly added to hydrochloric acid (25 mL). Then, 5 mL of DI water was added before Ti_3_AlC_2_ (1 g) was slowly added. The mixture was subsequently reacted for 24 h with stirring (800 rpm) at 35 °C. Finally, the MXene solution was centrifuged at 3500 rpm for 5 minutes, and this step was repeated several times until the pH was greater than 6. The resulting supernatant was collected for further testing and characterization. The concentration of MXene was determined by measuring 30 mL of the MXene suspension, filtering with filter paper, drying in a vacuum oven at 30 °C and weighing the filter paper before and after filtering. We took 4 centrifuge tubes (capacity of 50 mL) and filled them with 10 mL of the MXene aqueous solution (concentration of 1 mg/mL). Then, PVP powder was added at amounts of 0.015 g, 0.035 g, 0.045 g, and 0.06 g. The mixture was fully mixed with a vortex treatment, and then vacuum filtered to form an MXene/PVP filter paper membrane. The resistances of the MXene/PVP filter paper membrane were ~1.5 kΩ/sq, ~30 kΩ/sq, ~50 kΩ/sq and ~3.9 MΩ/sq, respectively.

### Preparation of PET-Cu interdigital electrodes and sensor arrays

Fabrication of a flexible electrode circuit board by the laser ablation strategy can be found in previous reports^32^. The PET-Cu interdigital electrode and sensor array electrode were fabricated by laser irradiation of the copper-clad PET film with a 3 W diode-pumped Nd: YAG laser.

### Characterization

The sheet resistance of the MXene/PVP filter paper membrane was measured by a four-point probe (SB120, Yangzhou Subo Electric Co., Ltd., Yangzhou, China). The sheet resistance of the MXene/PVP filter paper membrane was the average value of 10 measurements. The morphology and thickness of the sensor based on the MXene/PVP filter paper membrane, filter paper, pure MXene film, MXene/PVP filter paper membrane and elemental mapping were characterized by FE-SEM (ZEISS SUPRA55, Oberkochen, Germany). Optical microscopy images of the PET-Cu interdigital electrode were obtained by transparent reflectance polarizing microscopy (59XC-PC, Shanghai Optical Instrument Factory, Shanghai, China). XRD data of the MXene film, MXene/PVP film and filter paper were recorded using an X-ray diffractometer (D8 ADVANCE, Bruker, Germany). We used a computer-controlled stepper motor system to test the response of the sensor under static and dynamic pressures, including a force sensor (ZQ 990B, Dongguan Zhitou Precision Instrument Co., Ltd., Guangdong, China) and a TH2830 LCR meter (Changzhou Tonghui Electronic Co., Ltd., Jiangsu, China). The wireless sensor test (finger pressing) and part of the application test used the LZ-01 ARC(M) test system (Hangzhou Lingzhi Technology Co., Ltd., Hangzhou, China).

## Conclusions

In conclusion, we have developed a new method to fabricate capacitive pressure sensors with high sensitivity based on MXene/PVP and interdigital electrodes. The capacitance variation of the sensor is improved by adopting a parallel capacitor configuration and selecting an appropriate dielectric layer. The capacitive pressure sensor has a wide capacitance change ranging from ~3 pF to ~1000 pF. The capacitive sensor not only achieves high sensitivity (1.25 kPa^−1^) and a low detection limit (~ 0.6 Pa) but also has a wide sensing range (up to 294 kPa), fast response and recovery times (30/15 ms) and mechanical stability for 10000 cycles. The sensor can detect a wrist pulse, human swallowing, human respiration and voice recognition. Our capacitive array sensors have been successfully used to map the surface pressure distribution of coins with different weights along with a key. The sensor is made of an interdigital electrode, which demonstrates easy integration, circuit design and application to distributed sensor arrays. Choosing the appropriate interdigital electrode and appropriate dielectric layer can reduce the size of the device, which is conducive to the preparation of a micro-capacitive sensor. We believe that by optimizing the structure and using a more ideal dielectric layer, the sensitivity of the sensor will be greatly improved. This method is simple and cost-effective, easy to transition into large-scale production and does not require complex processes or additional packaging. Furthermore, these sensors would have wide application prospects in wearable medical and health monitoring devices, intelligent robots and efficient human-machine interfaces.

## Supplementary information

SUPPLEMENTAL MATERIAL Information

The real-time response test of the sensor loading and unloading

The real-time response of the MXene/PVP-based capacitive pressure sensor
