# Supplementary material for: A new strategy for the fabrication of a flexible and highly sensitive capacitive pressure sensor
Source: Microsyst Nanoeng. 2021 Nov 30;7:100. doi: 10.1038/s41378-021-00327-1 (PMC8630520; doi:10.1038/s41378-021-00327-1)
Supplement: Supplementary file 1 — SUPPLEMENTAL MATERIAL Information [file 41378_2021_327_MOESM1_ESM.docx]

# Supplementary Information

##### A new strategy for fabrication of a flexible and highly sensitive capacitive pressure sensor

Ruzhan Qin^1^, Mingjun Hu^2^, Xin Li^1^, Te Liang^1^, Haoyi Tan^1^, Jinzhang Liu^2^, and Guangcun Shan^1^

^1^School of Instrumentation Science and Opto-electronics Engineering, Beihang University, Beijing 100191, China

^2^School of Materials Science and Engineering, Beihang University, Beijing 100191, China

Correspondence: Guangcun Shan (gcshan@buaa.edu.cn)


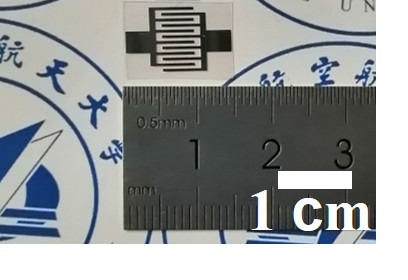


Fig. S1. Photograph of a PET-Cu interdigital electrode (12 fingers) prepared by laser ablation strategy.


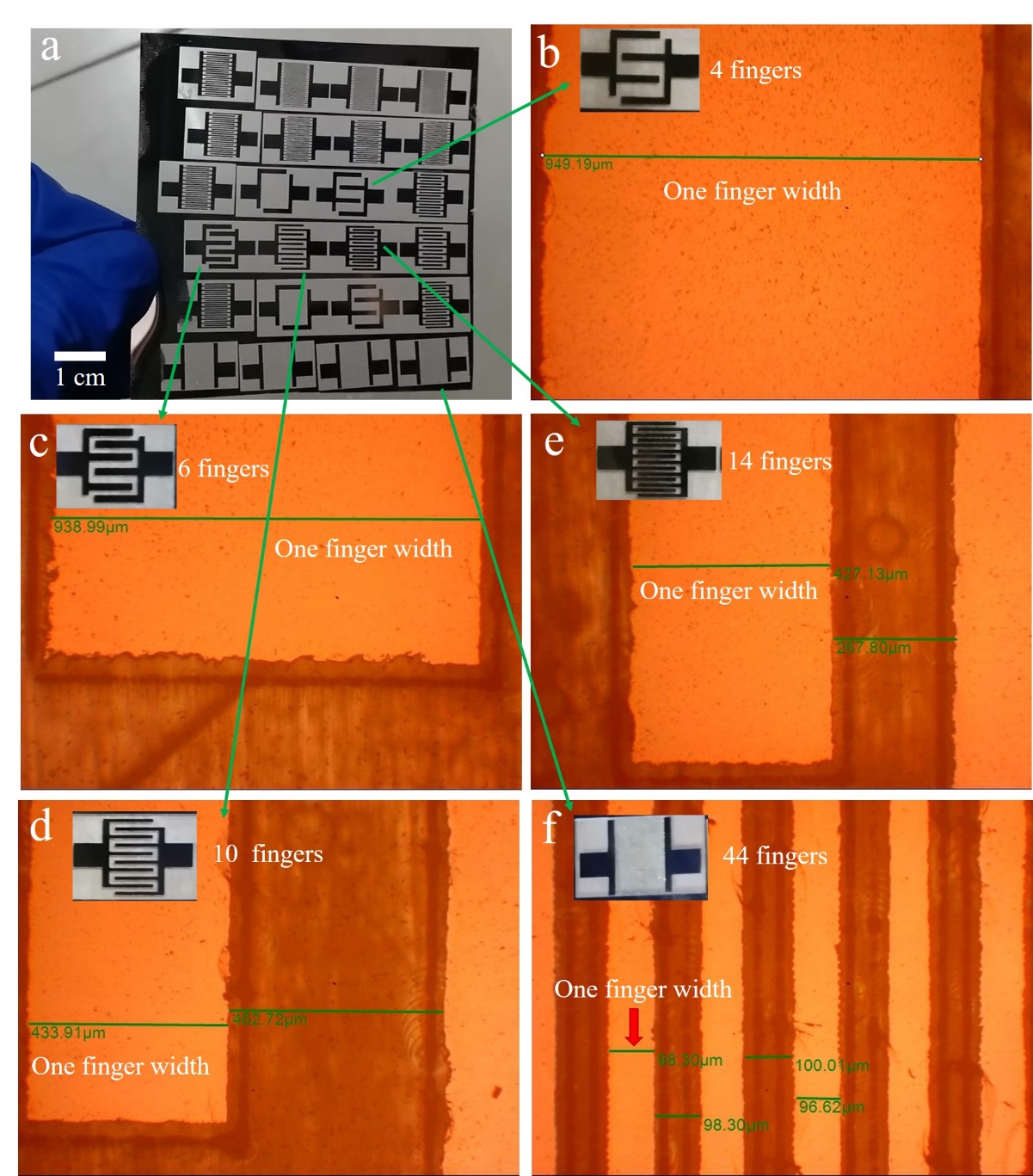


Fig. S2. (a) Photograph of PET-Cu interdigital electrodes with different number of interdigital fingers prepared by laser ablation strategy. (b-f) Optical microscope images of interdigital electrodes with 4, 6, 10, 14 and 44 fingers respectively.


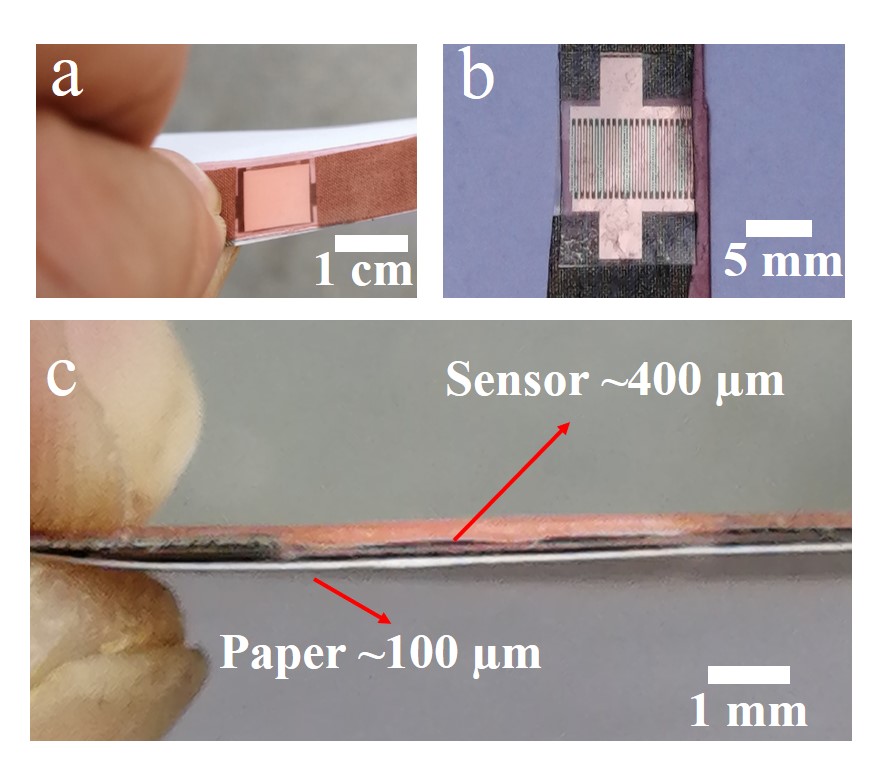


Fig. S3 (a-c) The front, back and cross-section of the prepared sensor (44 fingers). The cross-sectional view of the sensor is compared with the paper thickness.


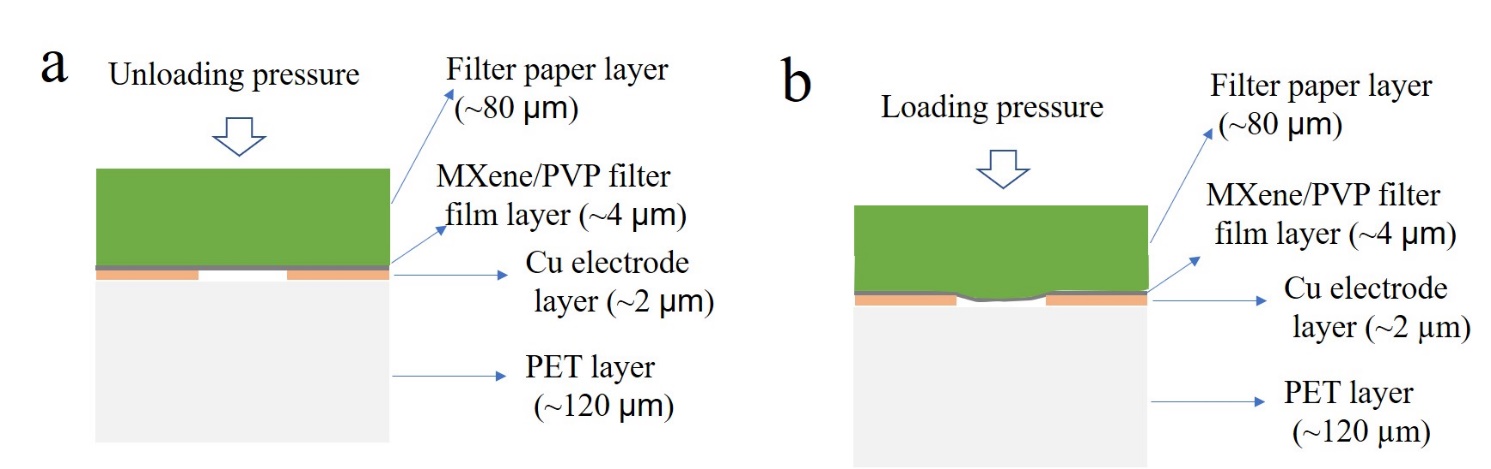


Fig. S4 (a) When the sensor is unloading pressure, the gap between two Cu electrode pairs is air medium. (b) When the sensor is loading pressure, part of the MXene/PVP filter paper membrane dielectric layer is filled into the gap between the two electrode pairs.


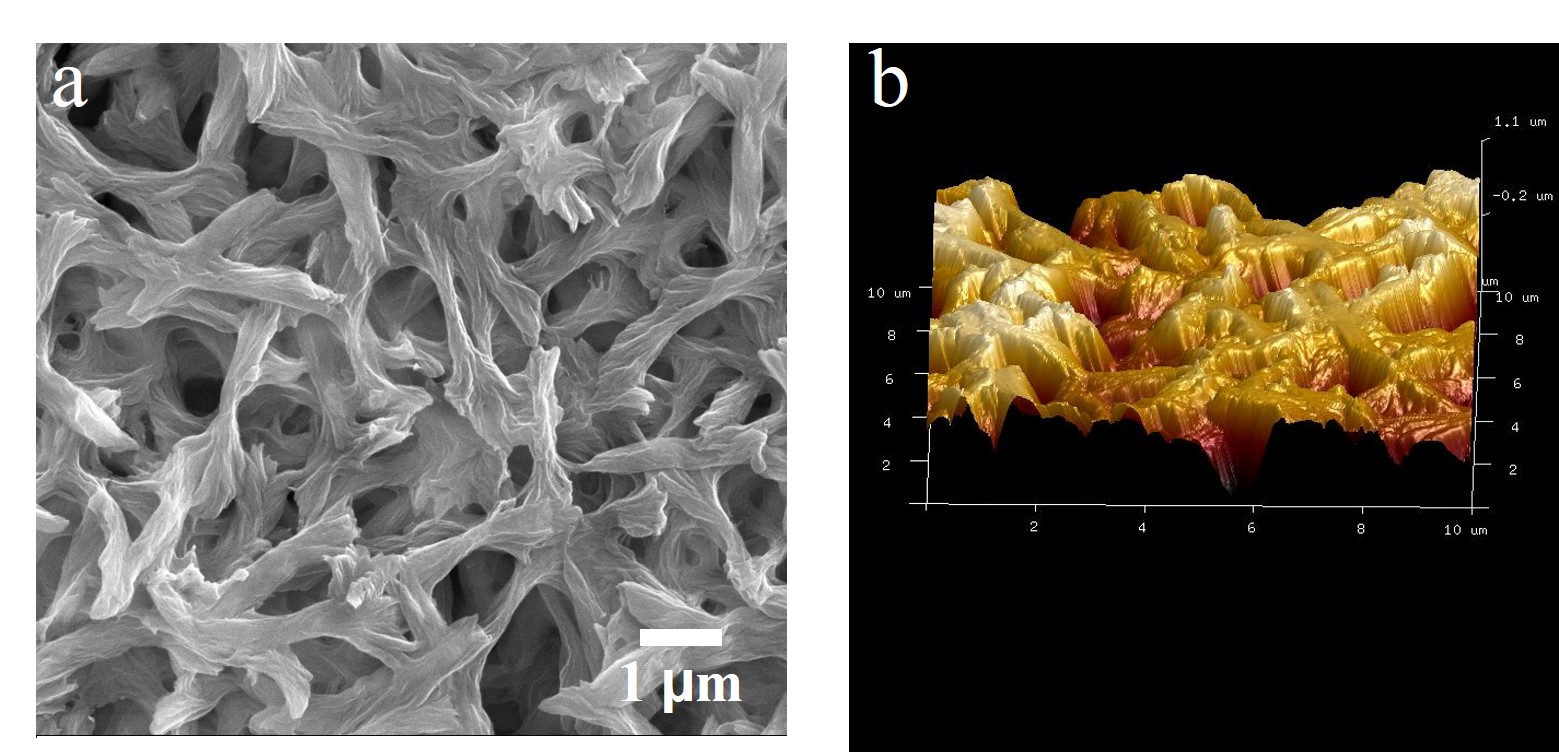


Fig. S5. (a) SEM image of porous and uneven structure on the surface of filter paper. (b) AFM image of porous and uneven structure on the surface of filter paper.


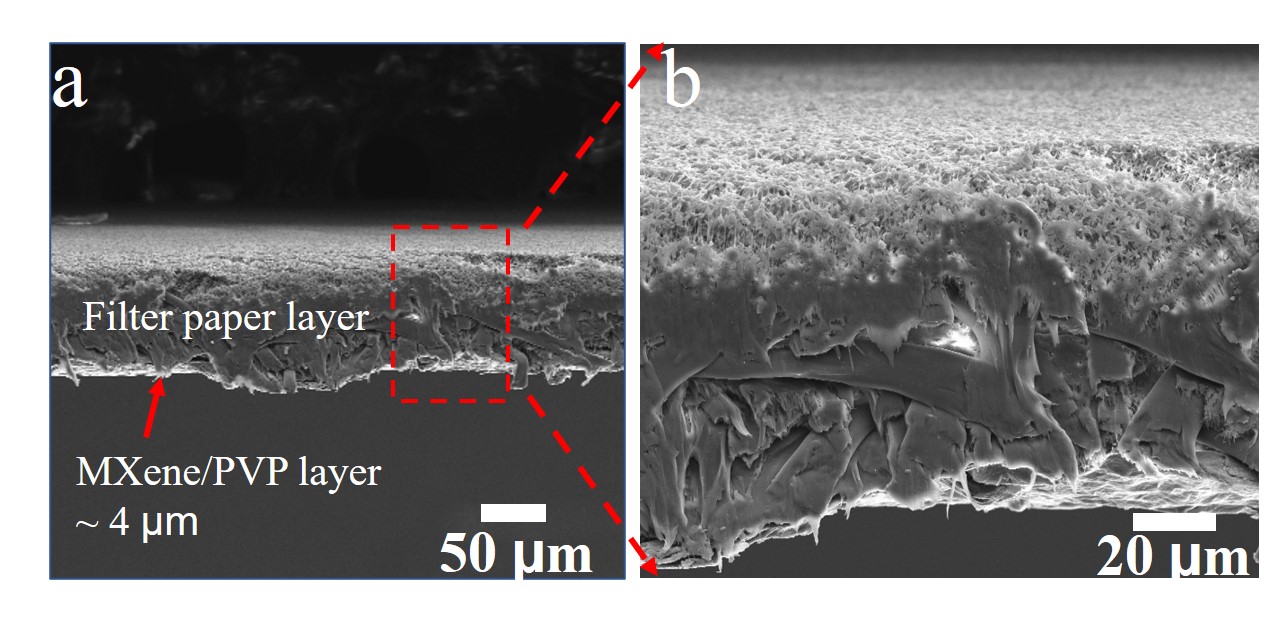


Fig. S6. (a-b) SEM images of the cross section of MXene/PVP filter paper and its local enlarged images.


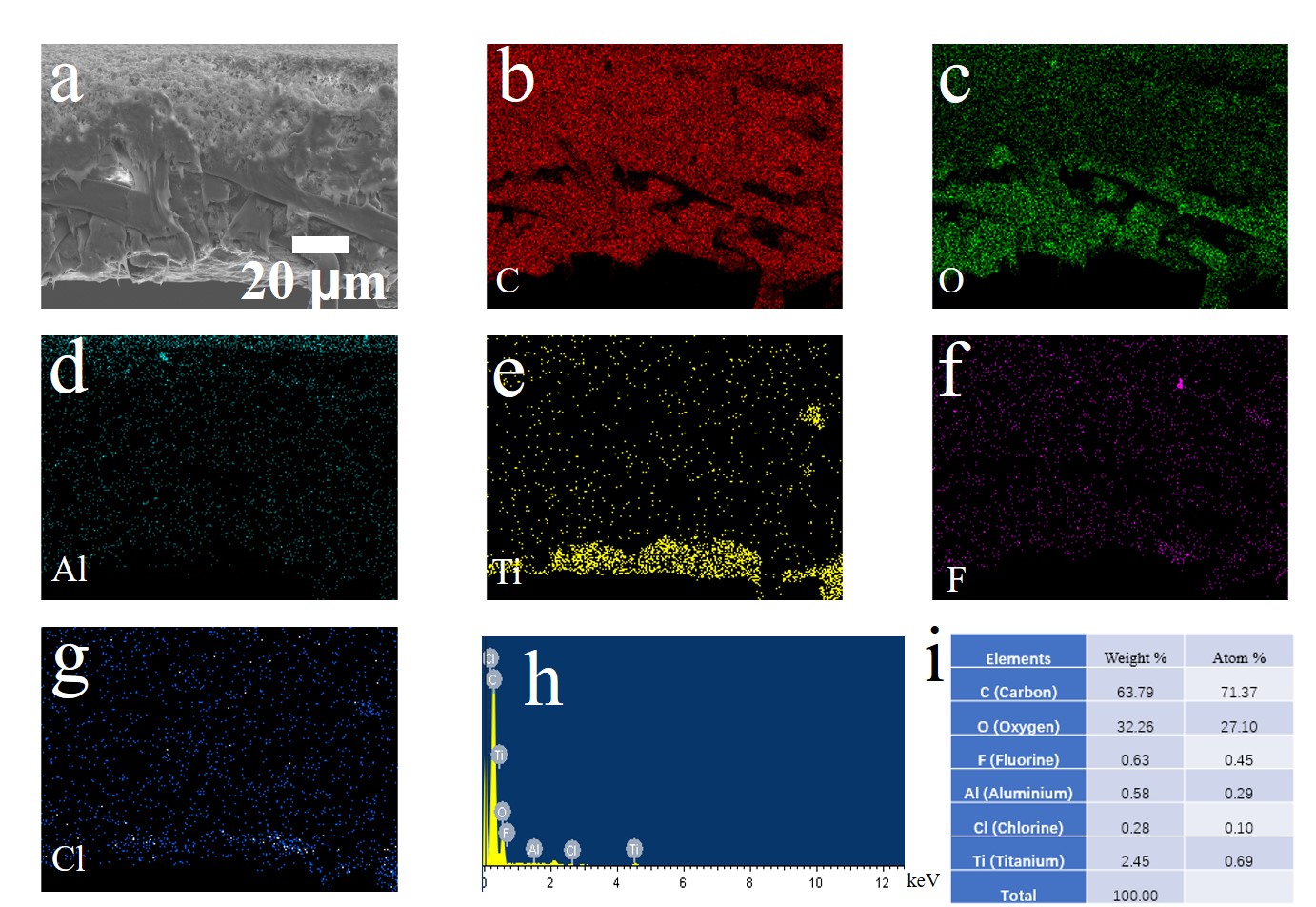


Fig. S7. (a) SEM image of the cross section of MXene/PVP filter paper membrane. (b-g) EDS element mappings of the cross section of MXene/PVP filter paper membrane. (h) Element spectrogram of the cross section of MXene/PVP filter paper membrane. (i) Weight and atomic percentage of elements in the cross section of MXene/PVP filter paper membrane.


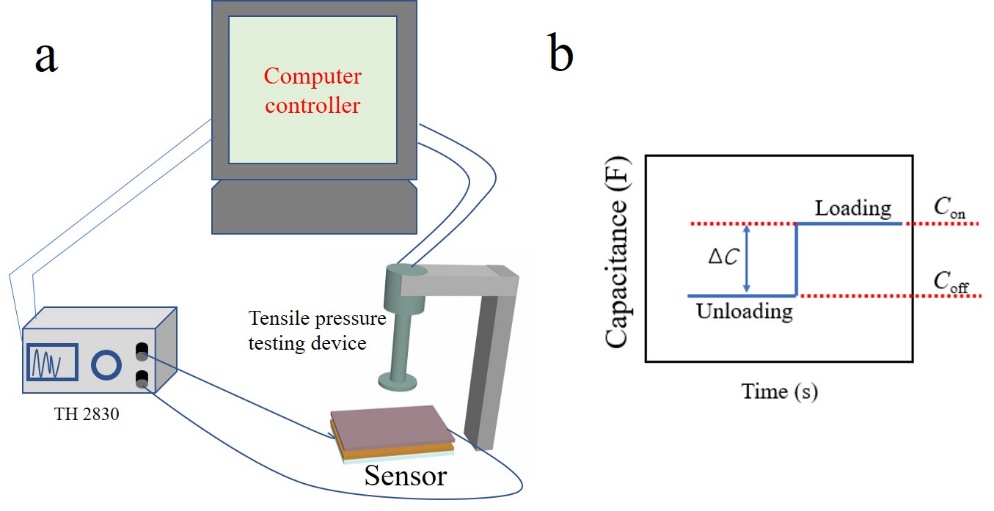


Fig. S8. The pressure testing device and associated source meter used to test sensor performance. (b) The testing principle of sensor performance.


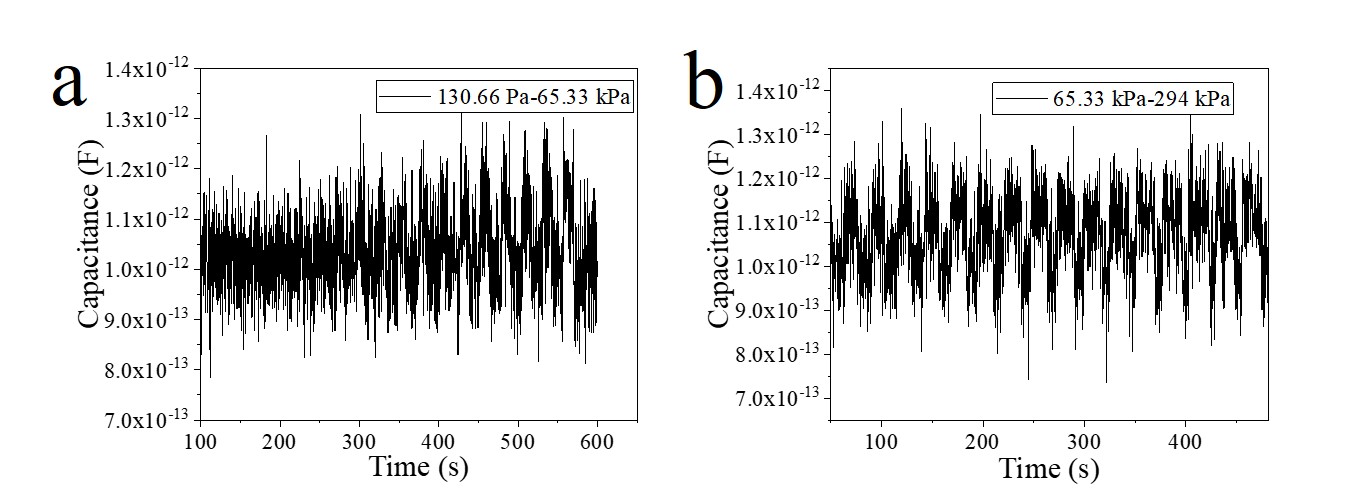


Fig. S9. (a-b) *C*-*T* curves of the sensor with PET single-sided adhesive tape (without MXene/PVP filter paper membrane) under different pressure.


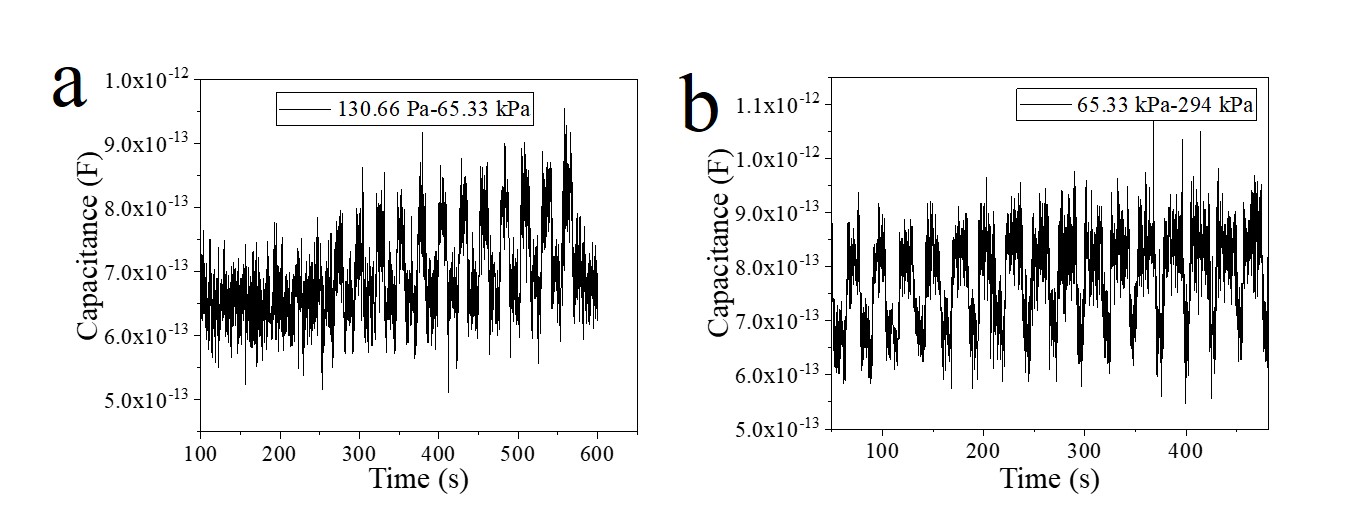


Fig. S10. (a-b) *C*-*T* curves of the sensor base on pure filter paper +PET single-sided adhesive tape layer (without MXene/PVP filter paper membrane) under different pressure.


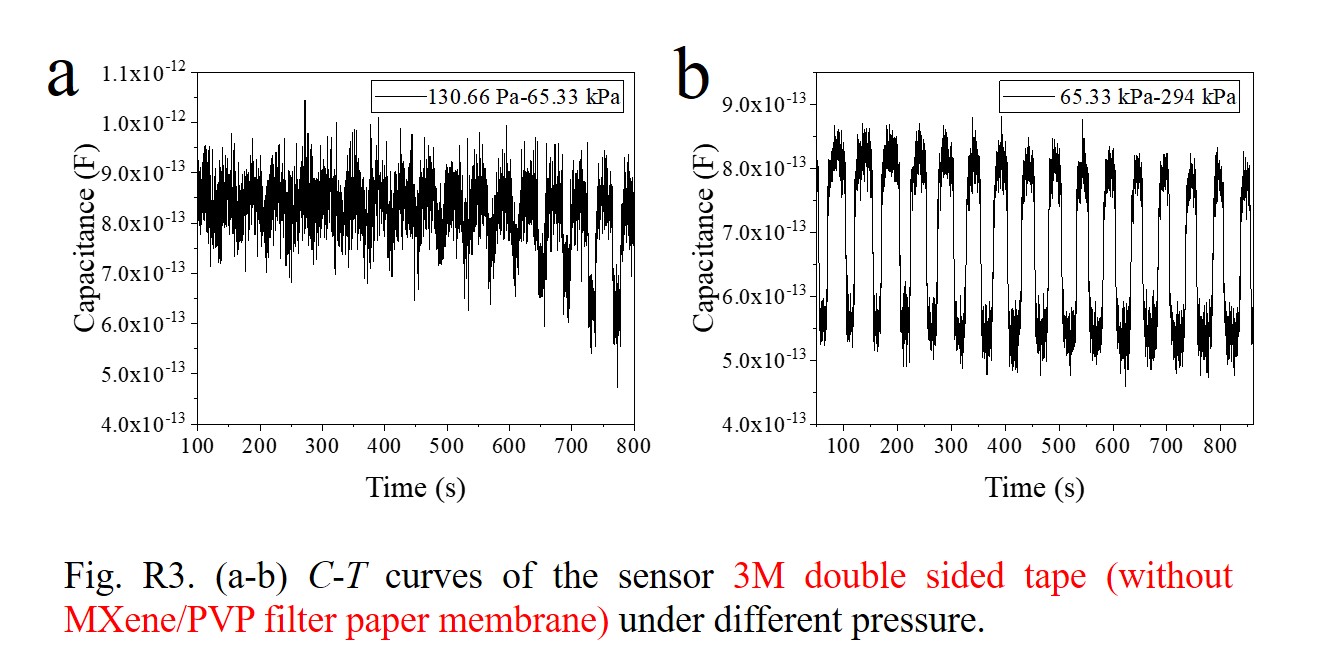


Fig. S11. (a-b) *C*-*T* curves of the sensor 3M double sided tape (without MXene/PVP filter paper membrane) under different pressure.


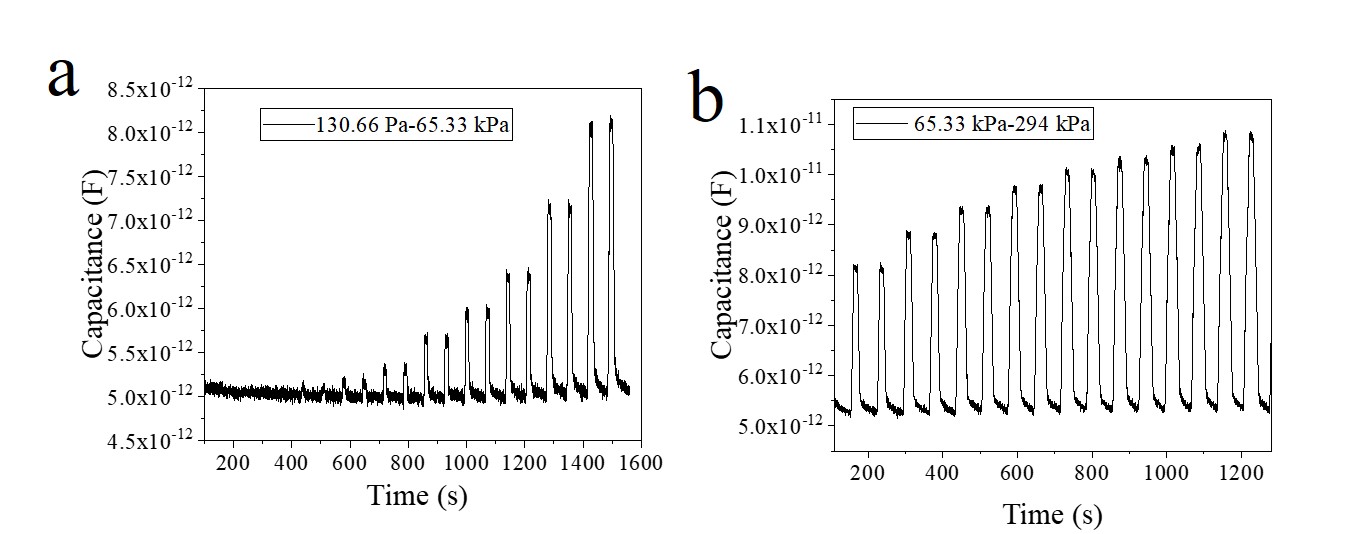


Fig. S12. (a-b) *C*-*T* curves of the sensor with MXene/PVA filter paper membrane (~50 kΩ/sq) under different pressure.


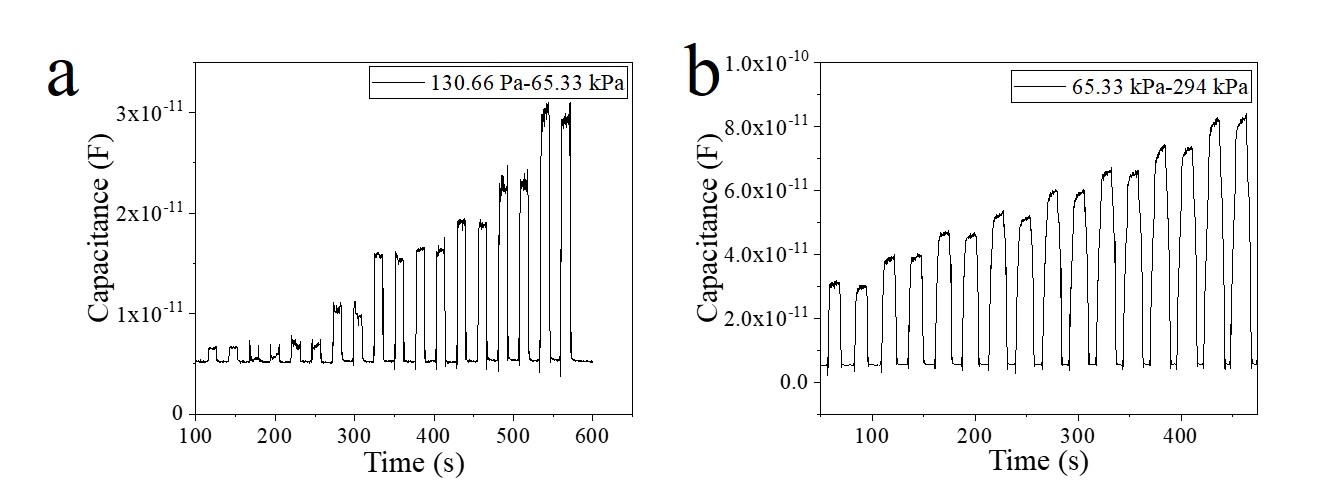


Fig. S13. (a-b) *C*-*T* curves of the sensor with MXene/PVP filter paper membrane (~50 kΩ/sq) and without PET single-sided adhesive tape layer under different pressure.


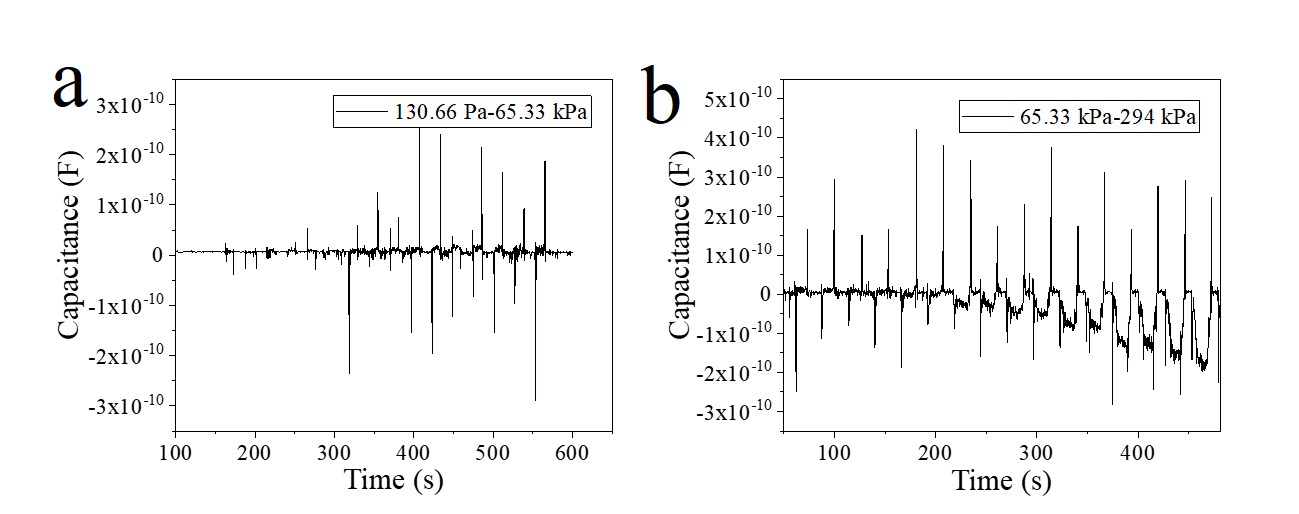


Fig. S14. (a-b) *C*-*T* curves of the sensor base on MXene/PVP filter paper membrane (~1.5 kΩ/sq) under different pressure.


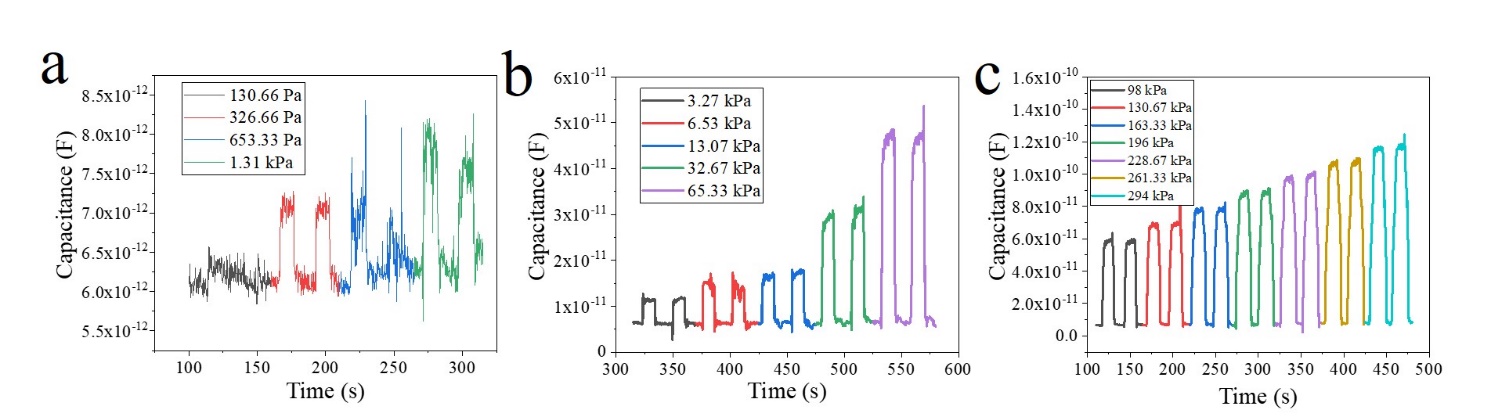


Fig. S15. (a-c) *C*-*T* curves of the sensor base on MXene/PVP filter paper membrane (~30 kΩ/sq) under different pressure.


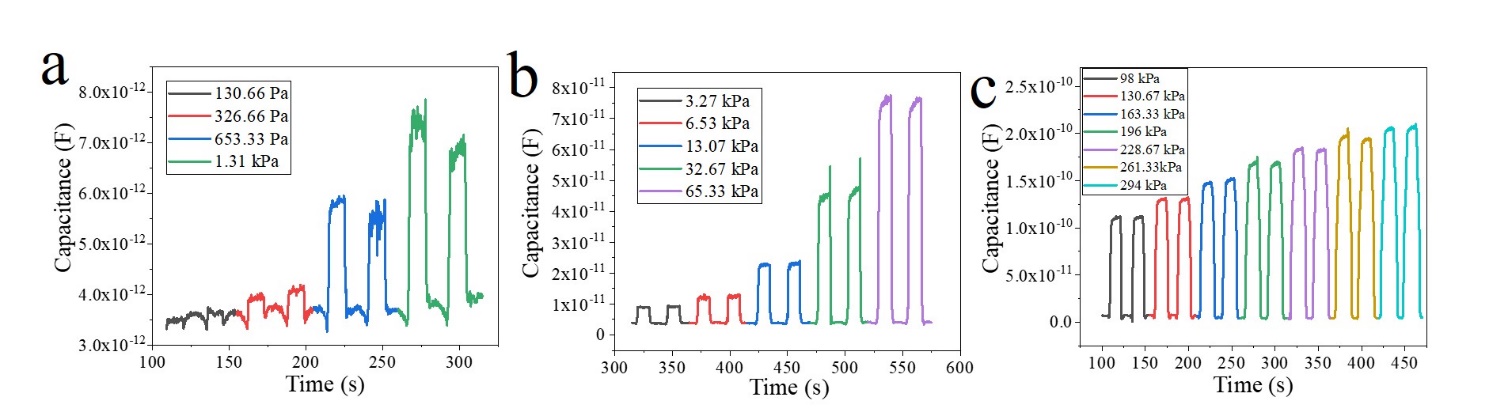


Fig. S16. (a-c) *C*-*T* curves of the sensor base on MXene/PVP filter paper membrane (~50 kΩ/sq) under different pressure.


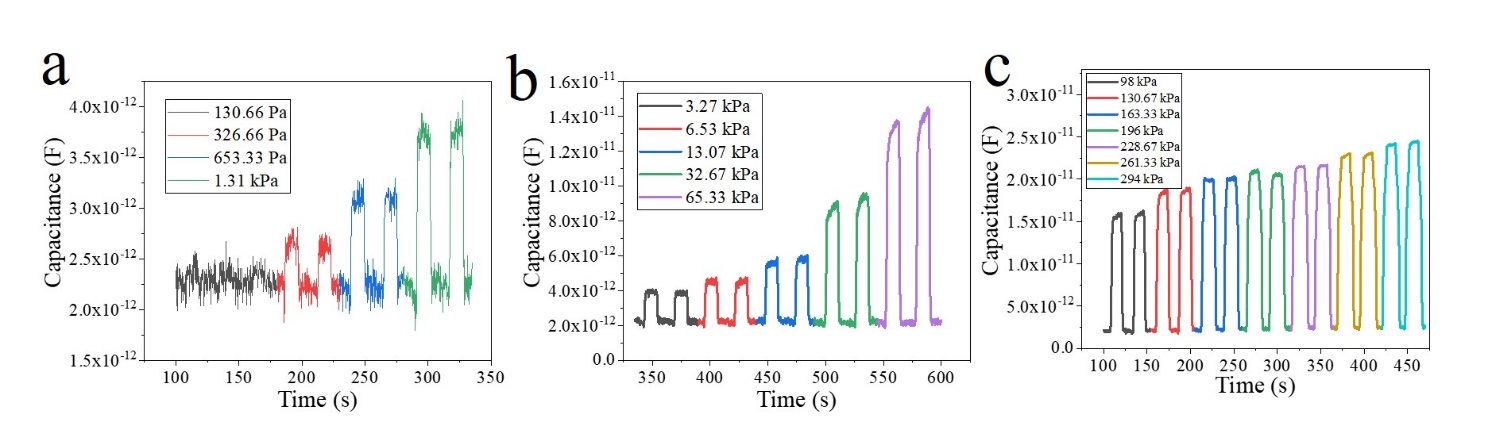
Fig. S17. (a-c) *C*-*T* curves of the sensor base on MXene/PVP filter paper membrane (~3.9 MΩ/sq) under different pressure.


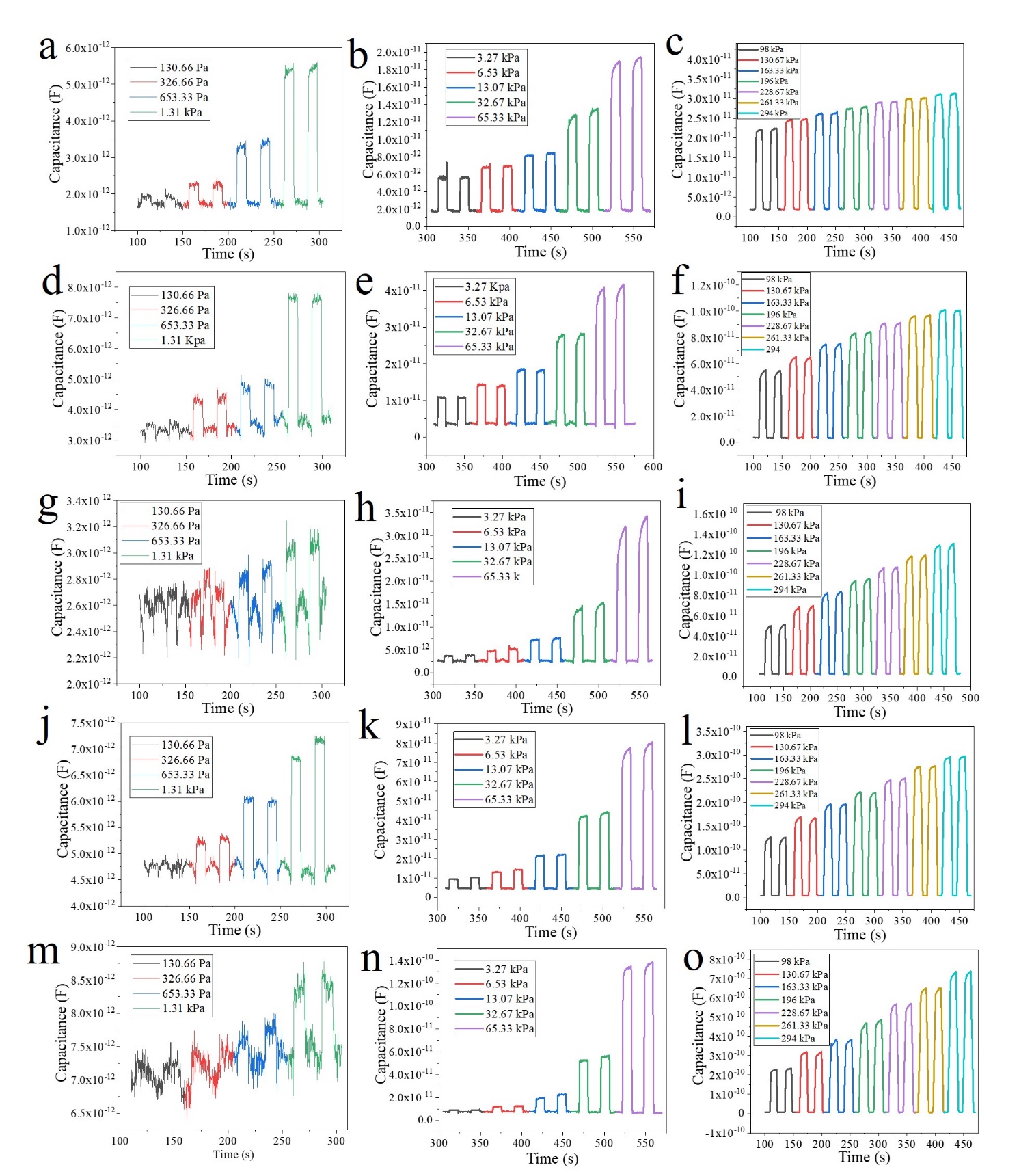


Fig. S18. (a-c) *C*-*T* curves of the sensor with 4 interdigital fingers under different pressure. (d-f) *C*-*T* curves of the sensor with 6 interdigital fingers under different pressure. (g-i) *C*-*T* curves of the sensor with 10 interdigital fingers under different pressure. (j-l) *C*-*T* curves of the sensor with 14 interdigital fingers under different pressure. (m-o) *C*-*T* curves of the sensor with 44 interdigital fingers under different pressure. (All above sensor is based on MXene/PVP filter paper membrane with resistance of ~ 50 kΩ/sq.)


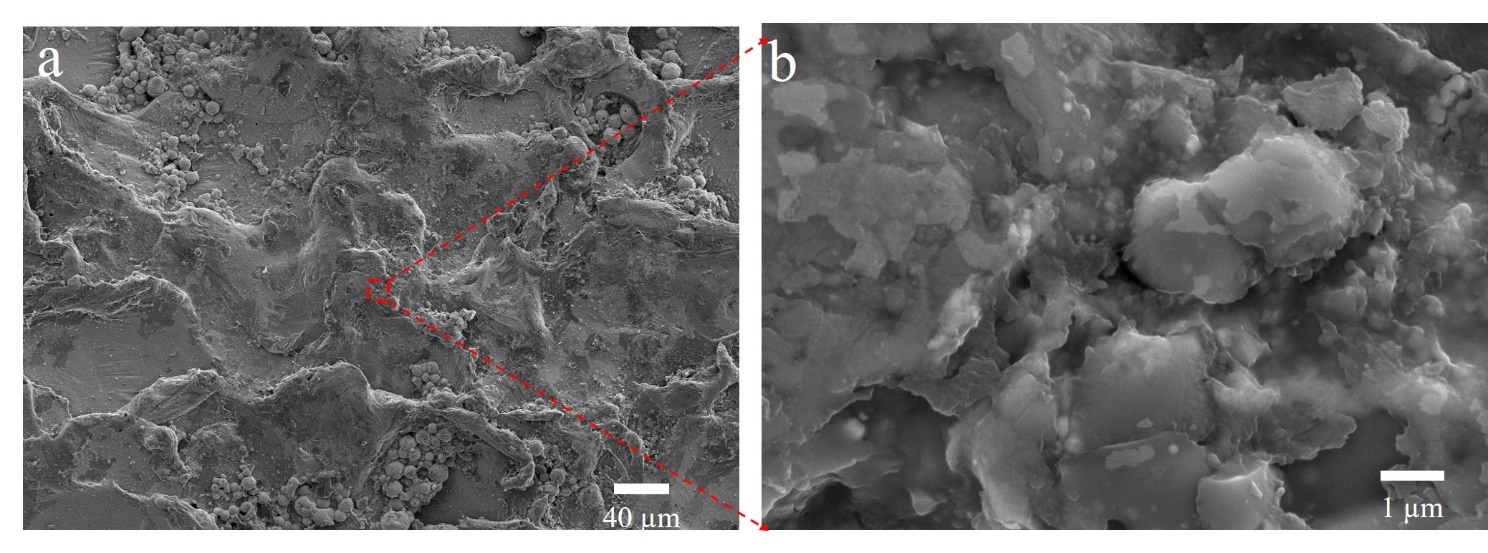


Fig. S19. (a-b) SEM image and its local enlarged images of the surface structure of PET single-sided adhesive tape with small elastic modulus.


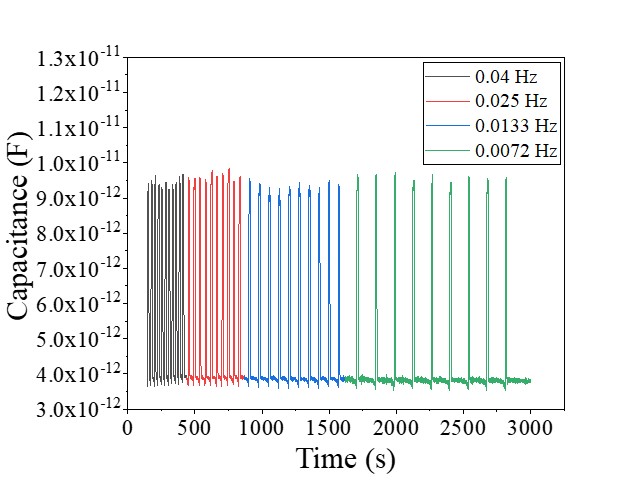


Fig. S20. Sensor frequency response curve.

Table S1. Summary and comparison of capacitive pressure sensor performance based on MXene or other material between this work and some papers published in recent three years.

| Materials | Detection  limit | Sensing range | Sensitivity | Response  time | Stability  (cycles) | References |
| --- | --- | --- | --- | --- | --- | --- |
| MXene/PVP | 0.6 Pa | < 294 kPa | 1.25 (130.66 Pa - 98 kPa)  0.88 (98 kPa - 294 kPa) | ~30 ms  /~15 ms | 10000 | This work |
| Graphene  /Ecoflex | 5 Pa | 5 Pa - 20 kPa | 0.13 kPa^−1^(< 0.1 kPa),  0.00441 kPa^−1^(>10 kPa) | 66 ms  /80 ms | / | [8] |
| MXene  /PVDF-TrFE | 1.5 Pa | 0 Pa -  400 kPa | 0.51kPa^−1^ (0 - 1 kPa),  0.011 kPa^−1^ (10 kPa - 150 kPa),  0.006 kPa^−1^ (150 - 400 kPa) | 150 ms  /150 ms | 10000 | [9] |
| Hybrid foam | / | 0 Pa -  240 kPa | 0.049 kPa^−1^ (0 - 240 kPa) | 55 ms  /160 ms | / | [11] |
| Icicle-shaped liquid metal | 12 Pa | 0 - 25 kPa | 0.39 kPa^−1^ (0 - 1 kPa),  0.15 kPa^−1^ (1 - 6 kPa),  0.1 kPa^−1^ (6 - 25 kPa) | 190 ms  /310 ms | 6000 | [13] |
| PDMS  /Scrubber | 4.4 Pa | 4.4 Pa - 216 kPa | 0.0046 Pa^−1^(4 Pa - 200 Pa),  0.0051 Pa^−1^(44 Pa - 2000 Pa),  0.000446 kPa^−1^(2.2 kPa - 120 kPa) | / | / | [15] |
| carbon fiber/  /PDMS | 1.2 Pa | 0 - 50 kPa | 0.82 kPa^-1^ (0 - 2 kPa),  0.13 kPa^-1^ (2 - 50 kPa) | 200 ms  /300 ms | 10000 | [16] |
| Carbon fiber/  Honeycomb fabrics | / | 0 - 40 kPa | 0.045 kPa^-1^ (0 - 10 kPa),  0.022 kPa^-1^ (10 kPa - 40 kPa) | 135 ms | 1000 | [17] |
| 3D AgNWs/ TPU | 0.9 Pa | 0 - 28 kPa | 1.21 kPa^−1^(0 - 3 kPa),  0.15 kPa^−1^(3 - 28 kPa) | 100 ms /100 ms | 10000 | [23] |
| Fiber/Silicone | / | 0 - 100 kPa | 0.0121 kPa^−1^(0 - 100 kPa) | / | 100 | [33] |

Movie S1:

The real-time response test of the sensor loading and unloading 5 cm × 5 cm paper (0.1607 g) confirmed that the detection limit of the sensor is 0.6 Pa.

Movie S2:

The real-time response of the MXene/PVP-based capacitive pressure sensor is realized by finger pressing, and the response signal is displayed on the mobile phone through wireless transmission.
